# Hospitals and a Paid Staff

**Published:** 1911-08-19

**Authors:** 


### The Hospital

A Journal of

The Medical Sciences and Hospital Administration.

New Series. No. 238, Vol. IX. [No. 1301, Vol. L.] SATURDAY, AUGUST 19, 1911

### HOSPITALS AND A PAID STAFF.

When a hospital receives State or municipal aid, the voluntary principle which underlies the hospital system in this country is to a certain extent modified. The essence of voluntaryism, as applied to hospitals, is personal service to the sick. Those who are unable to supply such service in their own persons, as the majority of hospital supporters are, do so to a certain degree vicariously by contributing towards the funds of the charities in which they are interested. Others, again, give a measure of their time, their experience, and their business or organising capacity to the hospitals by serving on the boards of management, on special committees, or on the general advisory board. The service thus rendered cannot easily be estimated in pounds, shillings, and pence, but it is none the less a tangible asset to the institution which no hospital in this country can afford to lose.

The medical staff attached to a voluntary hospital is in a different position. A medical man tries to get on a hospital staff because such a position gives him, in existing circumstances, a higher status in the profession which he would not be able otherwise to obtain. The keen competition for staff appointments which has been so much discussed of late is a sign of the fact that such professional status does exist, and that the majority of men who hold hospital appointments are content to make sacrifices in order to obtain a position which may or may not prove a commercial asset. It has been said that it should prove to be a commercial asset in every case, but no one who has any experience of hospital work will agree that this necessarily follows. A specialist's or consultant's practice depends largely on personal as well as professional qualifications, and an appointment on a large hospital, even with a medical school attached, will not ensure a living wage unless the holder himself is a keen and able worker. The young consultant, who is attached to a children's hospital, or in a junior capacity to a general hospital, very often has a hard and pitiful struggle before him to keep body and soul together with that amount of respectability which his position demands. He has to give his services freely to the institution to which he belongs; to attend whenever called upon to do so, whether day or night; and to work, if he is in any way conscientious and realises the obligations which devolved upon him when he accepted the position, in a manner far harder than anybody who has not gone through the same probationary ordeal can imagine. For all this he gets status and experience, both admittedly very valuable things in themselves, but neither of which can be translated into income unless he wins his own footing. The argument that so many hundreds of men are willing to work like this, to give their honest service to our voluntary institutions in order to acquire a status and some added experience, may be pushed too far. At present the hospitals do not get the best men on their staffs. They get some of the best, it is true, but the system that exists makes it possible for a type of man below the best, who has money and leisure and influence behind him, to oust the better man who cannot afford to wait, and who, through stress of circumstance, is forced into general practice. So much the better for general practice, no doubt, but there is an obvious other side to the shield. The hospitals, especially the teaching hospitals, in this country, are the cradle of the profession, and it is imperative that those who watch over the cradle should be the very best men who are obtainable. Once we accept the principle, acknowledged in every institution that receives State or municipal aid, that a good man is worth his keep and something more, we shall have advanced an appreciable step in the right direction.

We have repeatedly urged the advisability of hospital committees paying their medical staffs reasonable remuneration for their services, and especially the junior staff. We ought to establish the principle of payment by mutual arrangement without delay. It is the principle that counts, as Emerson said, not the isolated instance or the exception. Once the principle is accepted, it is possible for hospitals to insist upon the enforcement of the strictest economy, that a man may hold only one hospital appointment, and that the wasteful and unscrupulous division of energy, whereby a member of the staff of three or more hospitals fulfils his responsibilities indifferently at each, is mischievous and to be condemned. The fact that a hospital is a voluntary institution makes no difference to that principle, for the work that the staff gives is of a different kind from that which other voluntary workers supply. We have already admitted the justice of the principle in the case of other hospital workers, and it is not such a far stretch to apply it to the professional staff as well. When the institution receives payment from outside for work done by the staff, as is likely to be the case under the new system of insurance, and as is already the case so far as the hospital treatment of school children is concerned, it becomes imperative to apply it with justice and liberality all round.

